# A Comparative Study of Desarda’s Versus Lichtenstein’s Technique for Uncomplicated Inguinal Hernia Repair

**DOI:** 10.7759/cureus.23998

**Published:** 2022-04-09

**Authors:** Dhanashree Moghe, Ramlal Prajapati, Amay Banker, Monty Khajanchi

**Affiliations:** 1 General Surgery, Seth Gordhandas Sunderdas Medical College and King Edward Memorial (KEM) Hospital, Mumbai, IND

**Keywords:** mesh, groin hernia, lichtenstein repair, desarda repair, inguinal hernia

## Abstract

Purpose

Since mesh-related long-term morbidity like chronic groin pain and vas entrapment in patients with an inguinal hernia is a concern, tissue-based repairs should be revaluated. There have been few prospective studies comparing the outcomes of Lichtenstein's technique and Desarda's technique for the repair of uncomplicated inguinal hernias. So, we conducted this prospective study comparing the two techniques.

Methods

This is a single-center prospective observational study conducted for a period of one year (2019). The patients who underwent surgery for uncomplicated inguinal hernia either by Lichtenstein's technique or Desarda's technique were included in the study. The two techniques were compared with respect to recurrence rates, immediate postoperative pain, chronic groin pain, wound infection, and the time taken to return to activities of daily living (ADL).

Results

There was no significant difference in the recurrence rates, chronic groin pain, wound infection, or return to ADL between Lichtenstein's technique and Desarda's technique of inguinal hernia repair. The mean duration to return to ADL was lesser when patients underwent Desarda's repair though this difference was not significant.

Conclusion

Desarda's tissue repair was found comparable to Lichtenstein's mesh repair in terms of recurrence and postoperative morbidity, immediate postoperative pain, chronic groin pain, wound infection, and the time taken to return to ADL. Desarda's technique may be considered as an alternative to mesh-based repairs to avoid long-term mesh-related morbidity for uncomplicated indirect hernias in the younger population.

## Introduction

Inguinal hernias, because of their frequency, remain an important surgical problem. The estimated lifetime risk for inguinal hernia is 27% for males and 3% for females. The annual mortality ranges from 100-to 300 per 100,000 inguinal hernia patients [1].

In the European Hernia Society Guidelines (EHS), mesh-based techniques, Lichtenstein's technique, in particular, are recommended for the treatment of symptomatic primary inguinal hernia [2]. Mesh works as a mechanical barrier, but it does not provide a mobile physiologically dynamic posterior wall [3]. The synthetic prostheses can create new clinical problems, such as foreign body sensation in the groin, discomfort, and abdominal wall stiffness, which may affect the patient's everyday functioning. Surgical site infections are more frequent after mesh-based hernia repairs [4]. An intense chronic inflammatory process typically associated with a foreign body reaction around the mesh prosthesis may produce meshoma or plugoma tumors, the treatment of which becomes a new surgical challenge [5]. Additionally, chronic scarring may lead to vas deferens obstruction, resulting in decreased fertility rates and a dysejaculation syndrome. A study by Cocuzza et al. found prosthetic mesh to exert long-term deleterious effects on the vas deferens, causing azoospermia [6]. Due to the observed complications and postoperative dysfunctions, many investigators took to new hernia repair techniques.

Desarda's technique, presented in 2001, is a novel hernia repair based on the concept of providing a strong, mobile, physiologically active, and dynamic posterior wall [7]. Desarda argued that since the aging process is minimal in tendons and aponeurosis, the use of a strip of external oblique aponeurosis (EOA) is the best alternative to either mesh or the Shouldice repair. The author demonstrated that his repair was dynamic in nature due to the contractions of the external and internal oblique muscles, thereby converting the strip of EOA into a 'shield' to prevent re-herniation. He also showed that the strip of EOA supported the transversalis fascia and that chances of herniation behind the strip were also reduced [7].

Since the original publication, few prospective studies have compared the outcomes of Lichtenstein's and Desarda's techniques for repairing uncomplicated inguinal hernias. So, we conducted this prospective observational study to compare the recurrence rates and the postoperative morbidity in terms of immediate postoperative pain, chronic groin pain, wound infection, and the time taken to return to activities of daily living (ADL) between Desarda's technique and Lichtenstein's technique for uncomplicated inguinal hernia.

## Materials and methods

Study design

We conducted a prospective observational study for a period of one year (2019) on the patients admitted to the department of general surgery in a single tertiary care hospital. The patients were operated on by multiple consultant surgeons with a minimum experience of one-year post-surgical training following the same operative steps as mentioned below. However, clinical evaluation at the specified follow-ups was performed solely by the first author. Approval of the Institutional Ethics Committee at Seth Gordhandas Sunderdas Medical College and King Edward Memorial (KEM) Hospital (protocol number EC/97/2018) was obtained.

Participants

After their informed consent, the patients who underwent surgery for uncomplicated inguinal hernia either by Lichtenstein's or Desarda's technique were included in the study. Individuals who underwent surgery for complicated inguinal hernia, irreducible, obstructed, strangulated inguinal hernia (diagnosis of the above-mentioned conditions would be clinical) were excluded from the study. These patients were followed up at definite intervals by the first author; postoperative day (POD) 1, POD 10, and six months post-surgery. From now on, those patients who have undergone inguinal hernia repair by Lichtenstein's repair will be referred to as the LR group, and those, who have undergone inguinal hernia surgery by Desarda's repair, will be referred to as the DR group.

Data collection

Patient records and operative notes were evaluated for demographic information and the type of procedure performed. Other data was collected using clinical evaluation at various follow-up intervals using validated scoring systems.

The outcome variables used to compare the two groups were hernia recurrence, wound infection, postoperative pain, and time to return to activities of daily living (ADL). The condition of the surgical wound was assessed clinically by localized tenderness, increased temperature, discharge, bruising, or tissue breakdown at POD 1, POD 10 (at the time of suture removal), and at six months (at follow-up visit). The severity of wound infection was graded using Southampton Scoring System (SSS), wherein grade 0 implies normal healing, grade 1 wounds have normal healing with mild erythema/bruising, grade 2 wounds have erythema plus other signs of inflammation, grade 3 wounds have haemoserous discharge, grade 4 wounds have purulent discharge, and grade 5 wounds are severe wound infections with tissue breakdown [8]. The ADL were defined as walking, bathing, dressing, household activities, and returning to work. The pain was measured using the visual analogue scale (VAS) postoperatively at the follow-up visits (on POD 1 and POD 10). Chronic groin pain was measured by VAS at six months. Postoperative recurrence of hernia was assessed clinically at the six-month follow-up.

Statistical analysis

Data was analyzed by SPSS for Windows (version 26.0; IBM Inc., Armonk, USA). A chi-square test was used to determine statistical significance for categorical data, and the unpaired t-test was used for continuous variables. Statistical significance was set at 0.05.

Operative technique

In all cases, pre-operative hair removal was performed, and a single dose of intravenous antibiotic (amoxicillin plus clavulanic acid, 1.2 mg) was administered prior to surgery. Lichtenstein's hernia repair was performed as described in the literature [9].

Desarda's repair was performed as follows. In this technique, operative steps up to herniotomy are carried out as usual. Then, the upper leaf of external oblique aponeurosis is sutured to the upturned part of the inguinal ligament using prolene or nylon interrupted sutures. The medial-most sutures are taken on the anterior rectus sheath, where the EOA is fused with it. After this, a strip of EOA is created by making an incision parallel to the inguinal ligament on the EOA. This splitting incision is taken to create a strip of EOA which is equivalent in width to the distance between the conjoined tendon and the upturned part of the inguinal ligament. This incision is extended from the pubic symphysis medially to just beyond the deep ring laterally. The upper border of this newly created strip is sutured to the inferior edge of the conjoined tendon using prolene or nylon sutures. This places the strip of EOA posterior to the cord, giving replacement to the absent aponeurotic element in the posterior wall of the inguinal canal. The newly created upper leaf of the EOA is then sutured to the lower leaf. Subcutaneous tissue and skin are closed using either simple sutures or skin staplers, depending on surgeon preference.

## Results

A total of 50 patients were included in this study. Each arm of the study had 25 patients, and the demographic characteristics of the two groups were comparable. The mean age of patients in group 1 was 27 years, and in group 2 was 28 years. Two patients in the DR group had a history of previous hernia repair. One underwent a right-sided herniotomy while the other patient had a left-sided open hernioplasty done. No patients in either group had a history of a chronic medical illness (Table 1).

**Table 1:** Patient demographics Categorical variables are written as count (percentage). Age is written as mean (± standard deviation). LR - Lichtenstein's repair; DR - Desarda's repair

	LR group (n=25)	DR group (n=25)	p-value
Age	27 (±4.2)	28 (±6.9)	0.886
Male sex	25 (100%)	25 (100%)	-
Hernia			
Direct	3 (12%)	5 (20%)	0.554
Indirect	22 (88%)	20 (80%)	0.766
Site			
Left	11 (44%)	10 (40%)	0.377
Right	14 (56%)	15 (60%)	0.763
Previous surgery	0	2 (8%)	-

The postoperative surgical site was assessed using the SSS. None of the patients in the study developed grade 2 or higher grades of wound infection. On POD 1, 10 (40%) patients in group 1 and nine (36%) patients in group 2 had grade 1 wound infection. On POD 10, eight and seven patients in group 1 and group 2, respectively, developed grade 1 wound infection. However, there was no significant difference between the groups as per the chi-square test (p>0.05; see Table 2).

**Table 2:** Surgical wound as per the Southampton score Categorical variables are written as count (percentage). POD - postoperative day; LR - Lichtenstein's repair; DR - Desarda's repair

	LR group (n=25)	DR group (n=25)	p-value
POD 1			
Grade 0	15 (60%)	16 (64%)	0.886
Grade 1	10 (40%)	9 (36%)	0.876
POD 10			
Grade 0	17 (68%)	18 (72%)	0.677
Grade 1	8 (32%)	7 (28%)	0.874
At six months			
Grade 0	25 (100%)	25 (100%)	-
Grade 1	-	-	-

The postoperative discomfort/pain was assessed by VAS score. At POD 1, 17 patients in group 1 and 16 patients in group 2 had VAS scores of 0-3, while three patients in both groups had VAS scores of 4-7. None of the patients in group 1 had a VAS score of 8-10, while one patient in group 2 had a VAS score of 8-10. At POD 10 and at six months, all patients in group 1 and group 2 had VAS scores of 0-3, and the mean VAS score was comparable between the groups (1.30±0.66 vs. 1.20±0.52). There was no significant difference between the groups (p>0.05; see Table 3).

**Table 3:** Assessment of postoperative pain using the visual analogue scale (VAS) Categorical variables are written as count (percentage) or mean (±standard deviation). POD - postoperative day; LR - Lichtenstein's repair; DR - Desarda's repair

	LR group (n=25)	DR group (n=25)	p-value
POD 1			0.296
0-3	17 (68%)	16 (64%)	
4-7	8 (32%)	7 (28%)	
8-10	-	2 (8%)	
Mean	2.55 (±0.89)	2.65 (±1.81)	
POD 10			-
0-3	25 (100%)	25 (100%)	
4-7	-	-	
8-10	-	-	
Mean	1.3 (±0.66)	1.2 (±0.52)	
At six months			-
0-3	25 (100%)	25 (100%)	
4-7	-	-	
8-10	-	-	
Mean	0.8 (±0.84)	1 (±0.7)	

The mean time taken to return to ADL was 1.90±1.02 days in group 1 and 1.53±0.84 days in group 2. There was no significant difference between the groups (p>0.05). There were no observed recurrences in either group during the follow-up period (Table 4).

**Table 4:** Postoperative outcomes Categorical variables are written as count (percentage) or mean (±standard deviation). ADL - activities of daily living; LR - Lichtenstein's repair; DR - Desarda's repair

	LR group (n=25)	DR group (n=25)	p-value
Duration of stay, days	1.12 (±0.33)	1.08 (±0.27)	0.988
Time to return to ADL, days	1.9 (±1.01)	1.52 (±0.84)	0.712
Recurrence	0	0	-

## Discussion

In our study, there was no significant difference in the recurrence rates, chronic groin pain, wound infection, or return to ADL between Lichtenstein's and Desarda's inguinal hernia repair techniques. The mean duration to return to ADL was lesser when patients underwent Desarda's repair though this difference was not significant.

The aim of a hernia repair surgery is to provide a strong, mobile, and physiologically dynamic posterior wall. The technique described by Dr. Desarda is a tissue-based repair where an undetached, movable aponeurotic strip of external oblique muscle is used that physiologically enforces the posterior wall of the inguinal canal [7]. Contraction of the external oblique muscle creates lateral tension in this strip, while contraction of the conjoined muscle pulls this strip upwards, creating tension superiorly, making the strip a 'shield' to prevent any herniation. This additional strength given by the aponeurotic strip to the posterior wall of the inguinal canal prevents herniation and is the essence of this operation. The tension created in this strip is graded as per the force of muscle contractions. Stronger intra-abdominal pressure, such as during coughing, results in stronger abdominal muscle contractions, and stronger muscle contractions result in increased tension in this strip. The strip or the suture line is without any tension at rest. Thus, a strong and physiologically dynamic posterior wall is prepared in this operation [10]. This could be one of the reasons for a reduced rate of hernia recurrence seen in this repair. This finding is supported by studies conducted by other authors, which also demonstrated that recurrence rates in Desarda's repair were similar to those of Lichtenstein's repair [10-12].

Our study showed no difference in the incidence of wound infections when patients were operated by Desarda's technique vs. Lichtenstein's technique for inguinal hernia repair. The time taken to return to ADL was also comparable between the two techniques. In a randomized controlled trial by Szopinski et al., there was no significant difference in the clinical outcomes observed during a three-year follow-up of adult male patients with a primary inguinal hernia operated by Desarda's or Lichtenstein's technique. Excluding seroma formation, the frequency of complications was also similar in the two groups [1]. In a study conducted by Dr. Desarda in 2008 comparing this technique with mesh-based repairs, he reported that patients in whom the author's technique was performed had a shorter hospital stay, less time to return to work, and fewer complications [11]. Manyilirah et al. also conducted a case-control study on Desarda's repair, comparing it to the Lichenstein's repair, and they showed that rates of wound infections in both the repairs were similar [10].

The postoperative assessment in our study showed comparable postoperative pain scores with the two techniques used to repair an inguinal hernia. In a study conducted by Youssef et al., 168 patients with primary, uncomplicated inguinal and inguinoscrotal hernias were randomly allocated into Desarda's or Lichtenstein's group (85 vs. 83, respectively) and followed up for two years. There was no significant difference in mean postoperative VAS scores for pain, foreign body sensation, and chronic groin pain [12]. 

However, Losanoff and Mills criticized the repair citing concerns for an incomplete and unreliable follow-up method [13]. Naguib et al. also objected to Desarda's repair claiming that suturing the EOA to the conjoined tendon does not strengthen the posterior wall. On the contrary, it disturbs the physiology of the abdominal wall as the muscles run in different directions in the inguinal canal region [14].

Our experience

We found that the learning curve to Desarda's technique was short, and the repair was easily reproducible by even the junior surgeons in our institute. The authors were worried about the anticipated tension on the EOA suture line, but we found no difficulty in approximating the EOA for inguinal canal closure after creating the undetached strip for strengthening the posterior wall. The mean age of patients operated by Desarda's technique in our study was 27 years, and many of the hernias were of an indirect variety. Since mesh-related morbidity such as chronic pain and/or entrapment of vas deference was a concern, our study cohort comprised younger patients with an indirect hernia. Based on our short experience with this technique, the authors felt that Desarda's repair was most suitable for uncomplicated indirect inguinal hernias, where it gave results equivalent to Lichtenstein's repair. The robustness of this repair for larger direct hernias with a lax posterior wall is yet to be evaluated.

Desarda's technique may be considered as an alternative to mesh-based repair to avoid mesh-related complications, particularly for uncomplicated indirect hernias in the younger population. This technique might be useful in cases with an infected field where the placement of mesh is hazardous. This can be addressed in a future study. 

Limitations

Our study is limited by small sample size and a short follow-up period. Lack of randomization may be responsible for a selection bias, and since the majority of the hernias were indirect in nature, the robustness of this repair for direct hernias with a lax posterior wall cannot be commented upon. The efficacy of this technique for cases of complicated or recurrent hernia repairs was not evaluated. The assessment of pain (VAS) and time to return to ADL, and recurrence of hernia may be subject to bias since their collection was not blinded. 

## Conclusions

Desarda's tissue repair was found comparable to Lichtenstein's mesh repair in terms of recurrence and postoperative morbidity, immediate postoperative pain, chronic groin pain, wound infection, and the time taken to return to ADL. Desarda's technique may be considered as an alternative to mesh-based repairs to avoid long-term mesh-related morbidity for uncomplicated indirect hernias in the younger population. 
